# A randomized clinical trial of isolated ambulatory phlebectomy versus saphenous thermal ablation with concomitant phlebectomy (SAPTAP Trial)

**DOI:** 10.1093/bjs/znac388

**Published:** 2022-12-05

**Authors:** Eveline R Y Scheerders, Simone K van der Velden, Lucas M A Goossens, Sterre A S Hamann, Marianne G R de Maeseneer, Wendy S J Malskat, Linda de Mik, Tamar E C Nijsten, Renate R van den Bos, the members of the SAPTAP group, E R Y Scheerders, S A S Hamann, W S J Malskat, M G R Maeseneer de, R R Bos van den, S K Velden van der, L Mik de, M T W Gaastra, S Koppen, K P Roos De, N H Shadid, O Wolff

**Affiliations:** Department of Dermatology, Erasmus MC University Medical Centre Rotterdam, Rotterdam, the Netherlands; Department of Dermatology, MohsA Clinics Skincentre, Eindhoven, the Netherlands; Erasmus School for Health, Policy and Management, Erasmus University Rotterdam, Rotterdam, the Netherlands; Department of Dermatology, Erasmus MC University Medical Centre Rotterdam, Rotterdam, the Netherlands; Department of Dermatology, Erasmus MC University Medical Centre Rotterdam, Rotterdam, the Netherlands; Department of Dermatology, Erasmus MC University Medical Centre Rotterdam, Rotterdam, the Netherlands; Department of Dermatology, Isala Zwolle, Zwolle, the Netherlands; Department of Dermatology, Erasmus MC University Medical Centre Rotterdam, Rotterdam, the Netherlands; Department of Dermatology, Erasmus MC University Medical Centre Rotterdam, Rotterdam, the Netherlands

## Abstract

**Background:**

Current treatment of patients with saphenous trunk and tributary incompetence consists of truncal ablation with concomitant, delayed or no treatment of the tributary. However, reflux of the saphenous trunk may be reversible after treatment of the incompetent tributary. The aim of this study was to determine whether single ambulatory phlebectomy with or without delayed endovenous truncal ablation (SAP) is non-inferior to thermal endovenous ablation with concomitant phlebectomy (TAP), and whether SAP is a cost-effective alternative to TAP.

**Methods:**

A multicentre, non-inferiority RCT was conducted in patients with an incompetent great saphenous vein or anterior accessory saphenous vein with one or more incompetent tributaries. Participants were randomized to receive SAP or TAP. After 9 months, additional truncal treatment was considered for SAP patients with remaining symptoms. The primary outcome was VEnous INsufficiency Epidemiological and Economic Study Quality of Life/Symptoms (VEINES-QOL/Sym score) after 12 months. Secondary outcomes were, among others, cost-effectiveness, perceived improvement of symptoms, and anatomical success.

**Results:**

Some 464 patients received the allocated treatment (SAP 227, TAP 237). VEINES-QOL scores were 52.7 (95 per cent c.i. 51.9 to 53.9) for SAP and 53.8 (53.3 to 55.1) for TAP; VEINES-Sym scores were 53.5 (52.6 to 54.4) and 54.2 (54.0 to 55.6) respectively. Fifty-eight patients (25.6 per cent) in the SAP group received additional truncal ablation. Treatment with SAP was less costly than treatment with TAP.

**Conclusion:**

One year after treatment, participants who underwent SAP had non-inferior health-related quality of life compared with those who had TAP. Treatment with SAP was a cost-effective alternative to TAP at 12 months.

**Registration number:**

NTR 4821 (www.trialregister.nl).

## Teaser

This multicentre non-inferiority RCT compared isolated ambulatory phlebectomy with or without delayed truncal ablation (SAP) and endovenous truncal ablation with concomitant phlebectomy (TAP). Based on disease specific health-related quality-of-life scores, SAP was non-inferior to TAP after 12 months’ follow-up. Treatment with SAP was shown to be a cost-effective alternative to TAP.

## Introduction

Chronic venous disease (CVD), in particular varicose veins, is common in Western countries, affecting about 25–40 per cent of the general population^1–3^. Varicose veins are usually due to superficial venous incompetence, which may involve the saphenous veins (great saphenous vein, GSV; anterior accessory saphenous vein, AASV; small saphenous vein, SSV), their tributaries, or both. The number and extent of varicose tributaries vary considerably between patients presenting with varicose veins. Approximately 30 per cent of incompetent GSVs are accompanied by one or more large incompetent tributaries needing treatment^4^.

Since the introduction of high ligation and stripping, treatment strategies for superficial venous incompetence have mainly been based on the classical descending pathophysiological concept of the origin of varicose veins. This concept states that superficial venous incompetence starts at an escape point (the saphenofemoral junction (SFJ), saphenopopliteal junction or perforating veins) and progresses further downwards to the trunk(s) and subsequently their tributaries. The current management strategy for patients with both saphenous trunk incompetence and one or more incompetent tributaries is, therefore, treatment of the GSV, AASV or SSV with concomitant, delayed, or no treatment of the incompetent tributaries with phlebectomy or ultrasound-guided foam sclerotherapy (UGFS)^5^. There is, however, also evidence that varicose vein development may be based on an ascending or multifocal evolution of reflux^6–10^. This theory implies that incompetence starts with changes within the venous wall and valves, occurring first at the level of the tributaries, followed by the saphenous trunks and then eventually the junction.

The latter theory resulted in the development of the ambulatory selective varices ablation under local anaesthesia (ASVAL) method, in which the incompetent tributaries are treated by means of phlebectomy, with preservation of the saphenous trunk^9,11,12^. By removing the tributary first, blood volume through the saphenous trunk will be reduced, leading to remodelling of the trunk^9,12^. In some patients, symptoms and saphenous trunk reflux persist after ASVAL, and in such patients the saphenous trunk can be treated in a second stage. A prospective study^6^ showed that treatment of patients presenting with GSV reflux and a large varicose GSV tributary by means of isolated ambulatory phlebectomy resulted in abolishment of reflux in half of the patients at 1-year follow-up. Symptoms had resolved in two-thirds of the patients.

These findings changed perspective, suggesting that incompetence of a saphenous trunk may be reversible in selected patients and that treating varicose veins in a more conservative manner by ASVAL may be a cost-effective strategy. To date, there is still a lack of evidence to support the use of ASVAL. Therefore, this treatment strategy received only a class IIb recommendation in the recently published European Society for Vascular Surgery guidelines on CVD^5^. In an effort to increase the level of evidence, an RCT comparing single ambulatory phlebectomy with or without delayed saphenous trunk ablation (SAP), with thermal ablation with concomitant phlebectomy (TAP)—the SAPTAP trial—was set up in patients with symptomatic GSV or AASV incompetence, and one or more incompetent tributaries. The term single ambulatory phlebectomy was used to indicate the procedure of isolated ambulatory phlebectomy. The aim of this RCT was to determine whether treatment with SAP is non-inferior to TAP, and to assess whether SAP is a cost-effective alternative to TAP.

## Methods

Patients with CVD referred to seven medical centres in the Netherlands were screened for eligibility. Eligible were adults (aged at least 18 years) with symptoms of CVD, clinically obvious varicose veins (clinical class at least C2, according to the Clinical Etiologic Anatomic Pathophysiologic (CEAP) classification^13^), reflux of the GSV or AASV on duplex ultrasosonography (DUS), defined as retrograde flow of 0.5 s or more after distal augmentation^5^, and refluxing varicose tributaries directly connected to the incompetent saphenous trunk at the level of the thigh and/or knee (not lower than 5 cm below the knee). The refluxing segment of the saphenous trunk had to be at least 5 cm long, the diameter of the GSV or AASV over 3 mm (measured at mid-thigh level for the GSV, and 3 cm below the SFJ for the AASV) and tributaries had to be considered suitable for phlebectomy (in practice visible and/or palpable and with a diameter of at least 3 mm).

Exclusion criteria were: bilateral GSV or AASV incompetence, previous treatment of the ipsilateral GSV (in the case of the AASV, previous treatment of the ipsilateral AASV) or tributaries, incompetence of the GSV or AASV in the same leg, previous ipsilateral high ligation of the SFJ, acute deep or superficial vein thrombosis (SVT), agenesis of the deep venous system or any other vascular malformation, post-thrombotic syndrome, pregnancy, immobility, arterial insufficiency (ankle : brachial pressure index below 0.6), or inability to understand the patient information leaflets and questionnaires.

Included patients were randomized to either the SAP group, in which patients received only ambulatory phlebectomy, or the TAP group, in which patients underwent combined endovenous laser ablation (EVLA) and phlebectomy. Additional treatment was considered in the SAP group if symptomatic patients showed persisting GSV/AASV reflux at 9 months’ follow-up. Patients were randomized using an automated randomization service operated by Erasmus Clinical Trial Centre, stratified by the medical centre.

Because of the technical differences in treatment procedures, it was not possible to blind the treating physicians. Owing to the noises and the materials used, and the typical side-effects that occur during or after the EVLA (barbecue taste in the mouth and burning sensation over the treated segment), blinding of the patients was also not possible. Assessors were not blinded for practical reasons.

All patients signed an informed consent form, after having received all the specific information regarding the SAPTAP trial. Patients randomized to the SAP group agreed to have an extra follow-up evaluation after 9 months and, if necessary, additional EVLA or an alternative treatment of the GSV or AASV.

### Ethics and registration

This trial was approved by the Medical Ethics Committee of Erasmus Medical Centre Rotterdam (MED-2014-334) and was registered in the Dutch Trial Register (NTR 4821).

### Preoperative evaluation and interventions

Basic demographics, BMI, clinical class (C) of the CEAP classification, and the revised Venous Clinical Severity Score (VCSS)^14^ were registered. Patients were asked to complete the EuroQol EQ-5D-3L™ questionnaire (http://www.euroqol.org), a generic health-related quality of life (HRQoL) questionnaire including a visual analogue scale (VAS) registering overall health experienced on a scale from 0 to 100, and the VEnous INsufficiency Epidemiological and Economic Study Quality of Life/Symptoms (VEINES-QOL/Sym)^15,16^, a validated disease-specific HRQoL questionnaire for patients with CVD.

Before the intervention, with the patient standing, all varicose veins were carefully marked on the skin and DUS (CX50, transducer L12-3; Philips, Bothel, WA, USA) was performed to guide marking of the incompetent GSV or AASV, and its connection with the superficial varicosities. Both types of procedure were carried out under tumescent anaesthesia in an outpatient setting. Prophylactic low molecular weight heparin was not administered routinely.

#### Isolated ambulatory phlebectomy

Small stab incisions were made over the varicose tributaries, after which the veins were exteriorized with a phlebectomy hook and divided. The cranial connection with the GSV/AASV was ligated using Vicryl^®^ 3.0 (Ethicon, Somerville, NJ, USA). Further phlebectomies were performed along the course of the refluxing tributaries that had been marked before operation. Stab incisions were closed with adhesive strips. At the end of the procedure, the total length of removed varicose tributaries was noted, specifying whether this was less than 15, between 15 and 30, or more than 30 cm.

#### Thermal ablation and concomitant phlebectomy

Venous access of the refluxing trunk was obtained by ultrasound-guided puncture at the most distal point of reflux (not lower than mid-calf). Subsequently, a 5-Fr sheath was inserted over a guidewire. The laser fibre was then inserted through the sheath and the laser tip positioned 1–2 cm from the SFJ, distally from the inferior epigastric vein. Tumescent anaesthetic solution was injected under ultrasound guidance. After activation, the laser fibre was pulled back continuously, delivering approximately 60 J per cm vein. Laser wavelengths of 940, 980 or 1470 nm, and radial, tulip or bare fibre tips were used. Subsequently, phlebectomies were performed during the same procedure as described previously. The length of saphenous trunk treated with EVLA was noted as well as the total length of removed varicose tributaries.

#### After treatment

After treatment, a medical elastic stocking was applied over local bandages. Patients were advised to wear this stocking for the first 48 h and, after removing the dressings, during daytime for at least 1 week. Patients were allowed to mobilize about half an hour after the procedure and resume normal activities as soon as possible.

### Follow-up and data-collection

Follow-up visits were scheduled at 3 months, 9 months (SAP group only), and 12 months after the initial treatment. During these visits, patients underwent physical examination and the postoperative VCSS was determined. DUS was performed to evaluate the anatomical result at the level of the treated GSV or AASV trunk, including the presence or absence of reflux^17^. Anatomical treatment success was defined as total obliteration of the trunk and/or absence of reflux in both groups. Partial obliteration of the lumen was described as partial recanalization; if a segment of the vein length was open, this was described as segmental recanalization. Partial or segmental recanalization in the absence of venous reflux was also considered to indicate successful treatment. An open or recanalized trunk with present reflux was considered to indicate unsuccessful treatment.

Quality of life was assessed at each follow-up visit, using the VEINES-QOL/Sym and EQ-5D™. Patient satisfaction with the treatment was also assessed, using a score from 0 (not satisfied at all) to 10 (most satisfied). An additional multiple-choice question addressed the perceived improvement after treatment; patients had to indicate whether they experienced no improvement at all, some improvement or major improvement. Postoperative complications were registered at 3 and 12 months (the latter only for patients in the SAP group who received additional treatment); postoperative bleeding, wound infection, nerve damage, deep vein thrombosis, pulmonary embolism, SVT, skin burn, and hyperpigmentation were recorded. During the extra follow-up visit at 9 months for the SAP group, additional truncal ablation was considered when patients had persisting or recurrent symptoms and persisting reflux of the GSV/AASV. The extra visit was set at 9 months, to give the saphenous vein enough time to remodel after treatment of the tributary.

### Outcomes

The primary outcome of this trial was the VEINES-QOL/Sym score at 12 months after the initial treatment.

If the confidence interval for the difference in VEINES-QOL/Sym scores between SAP and TAP fell within the predetermined margin of 5 per cent of the TAP group’s VEINES-QOL/Sym score at 12 months’ follow-up, SAP could be considered non-inferior to TAP.

Secondary outcomes were the proportion of patients in the SAP group who received additional treatment of the GSV/AASV, EQ-5D™ (including EQ-VAS) scores, patient satisfaction after treatment and perceived improvement, VCSS and anatomical success at 3- and 12-month follow-up, and cost-effectiveness.

### Statistical analysis

Normally distributed continuous variables are presented as mean (95 per cent c.i.) and the independent *t* test was used to compare groups. Categorical data were analysed using the χ^2^ test.

The non-inferiority limit was calculated on the maximal acceptable loss of HRQoL defined by VEINES-QOL/Sym scores and was estimated at 5 per cent. Assuming that there was no difference between the standard TAP and SAP treatments, a total of 472 patients was required to achieve a power of 80 per cent with a one-sided α-level of 5 per cent. With an expected loss to follow-up of 10 per cent, a sample size of 260 patients per group was set. Owing to a much longer than expected recruitment period (4 instead of 2 years) and consequent long delay in presentation of the data, this sample size was reconsidered. At this point, 480 patients had already been included in the study, meaning that the sample size was already large enough to achieve 80 per cent power. Furthermore, there was a relatively low percentage of loss to follow-up. Therefore, it was decided by the research group that preemptive closure of the recruitment period and thus lowering the sample size was acceptable at this stage, and would not result in loss of statistical power.

Statistical analysis was undertaken using SPSS^®^ version 25.0 (IBM, Armonk, NY, USA) and Stata^®^ version 17.0 (StataCorp, College Station, TX, USA). The VEINES-QOL/Sym data are presented as a VEINES-QOL score and a VEINES-Sym score, indicating the HRQoL and severity of symptoms respectively. Primary VEINES-QOL/Sym scores were standardized, averaged, and subsequently transformed into *t* scores. The mean(s.d.) score for the sample was 50(10)^18^. The score and differences between scores do not have an absolute value and can only be interpreted within the sample. Higher scores indicate better health status (better quality of life and fewer symptoms). Missing values for HRQoL scores were replaced by the median of the completed items reported by an individual for that (sub)scale.

VEINES-QOL/Sym scores and patient satisfaction were analysed in one multilevel repeated-measures model. The model took into account that measurements within patients could be correlated and did not apply constraints to the structure of the co-variance matrix. The explanatory variables were time (measurement at baseline (reference category), 3 months, and 1 year) and interactions of treatment and time. The estimates of the treatment effects after 3 months and 1 year were represented by the coefficients of these interaction terms. The 95 per cent confidence intervals were calculated using bootstrapping. Missing data in the primary outcome (VEINES-QOL/Sym) and in patient satisfaction outcomes were handled by the multilevel repeated-measure models.

### Cost-effectiveness analysis

Costs of both treatments were approximated using data from the DIS database of the Dutch Healthcare Authority (https://www.opendisdata.nl). The costs of TAP treatment consisted of the costs of phlebectomy and EVLA. The costs of the SAP treatment consisted of the costs of phlebectomy, an extra DUS (for analysis at 9 months’ follow-up), and an additional truncal ablation for the proportion of patients who needed subsequent treatment.

In this trial, all patients in the SAP group had additional DUS at 9 months’ follow-up. In practice, not all patients would actually need this additional DUS examination (that is asymptomatic patients), so an assumption had to be made regarding the percentage of patients who would actually receive an additional DUS in clinical practice. This percentage was set at 1.5 times the number of patients who received additional treatment, based on the amount of patients with persisting symptoms.

The uncertainty around the estimates of the costs of the treatment was determined by means of bootstrapping and 95 per cent confidence intervals were used. The costs and effects were expressed in a cost-effectiveness plane. An intention-to treat-analysis was carried out. The CONSORT statement for non-inferiority trials was used as a guideline^19,20^.

## Results

### Participant flow and baseline characteristics

**Fig. 1:**
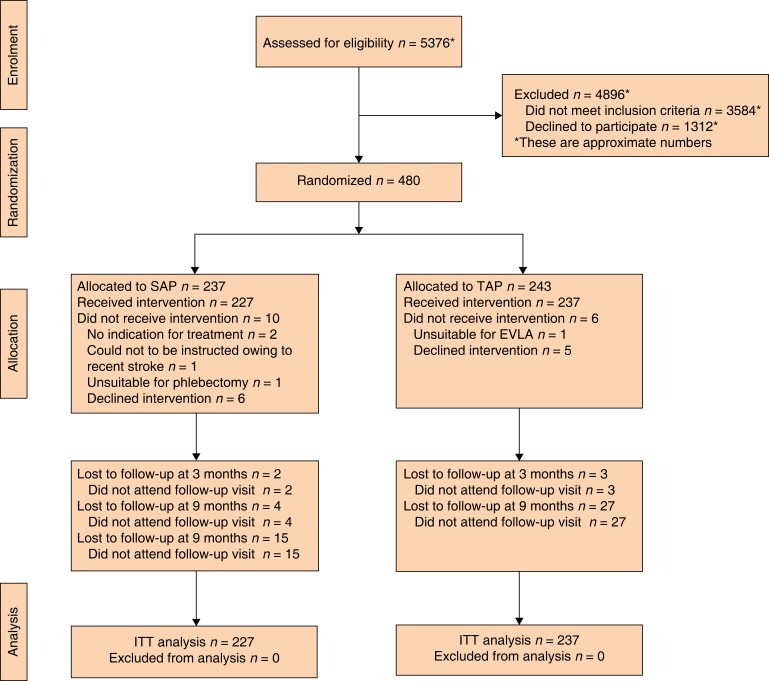
CONSORT diagram for trial SAP, isolated ambulatory phlebectomy with or without delayed endovenous truncal ablation; TAP, thermal ablation with concomitant phlebectomy; EVLA, endovenous laser ablation; ITT, intention to treat.

Between April 2015 and July 2019, some 480 patients met the criteria for eligibility and were randomized to either SAP or TAP. Of these, 464 patients received the allocated treatment. Sixteen patients were excluded because they no longer met the inclusion criteria (1 patient had a tributary unsuitable for phlebectomy, 1 patient appeared to have hypoplasia of the GSV in the thigh unsuitable for EVLA, 11 patients no longer wanted to participate, 1 patient could not be instructed sufficiently owing to a recent stroke, and in 2 patients there was no longer a clear indication for treatment) (*Fig. 1* and *Table 1*).

**Table 1:** Patient and treatment characteristics at baseline according to treatment received

	SAP (*n* =227)	TAP (*n* =237)
Age (years), mean(s.d.)	48.8 (14.0)	48.9 (14.4)
Sex ratio (M : F)	72 : 155	81 : 156
BMI (kg/m^2^), mean(s.d.)	27.4 (5.0)	26.9 (4.8)
SFJ reflux present	195 (85.9)	219 (92.4)
**Refluxing saphenous trunk**		
ȃGSV	145 (63.9)	147 (62.0)
ȃAASV	80 (35.2)	87 (36.7)
ȃMissing	2 (0.9)	3 (1.3)
VCSS at baseline, mean(s.d.)	6.0 (2.2)	5.9 (2.3)
**C(EAP) class**		
ȃC2	66 (29.1)	66 (27.8)
ȃC3	94 (41.4)	108 (45.6)
ȃC4–6	57 (25.1)	54 (22.8)
ȃMissing	10 (4.4)	9 (3.8)
**Total phlebectomy length (cm)**		
ȃ< 15	10 (4.4)	27 (11.4)
ȃ15–30	81 (35.7)	79 (33.3)
ȃ> 30	131 (57.7)	110 (46.4)
ȃMissing	5 (2.2)	21 (8.9)
Length of treated trunk (cm), mean(s.d.)	–	20.9 (10.7)
Energy used (J/cm), mean(s.d.)	–	60.6 (14.9)

Values are *n* (%), unless otherwise indicated. SAP, isolated ambulatory phlebectomy with or without delayed endovenous truncal ablation; TAP, thermal ablation with concomitant phlebectomy; SFJ, saphenofemoral junction; GSV, great saphenous vein; AASV, anterior accessory saphenous vein; VCSS, Venous Clinical Severity Score; CEAP, Clinical Etiologic Anatomic Pathophysiologic.

### Primary outcome: disease-specific health-related quality of life

**Fig. 2:**
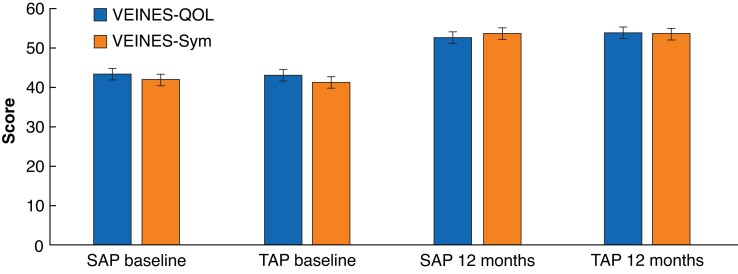
**VEINES-QOL/Sym scores at baseline and 12 months**’ **follow-up** Values are means with 95% confidence intervals. VEINES-QOL/Sym, VEnous INsufficiency Epidemiological and Economic Study Quality of Life/Symptoms; SAP, isolated ambulatory phlebectomy with or without delayed endovenous truncal ablation; TAP, thermal ablation with concomitant phlebectomy.

After 12 months’ follow-up, the VEINES-QOL/Sym scores for patients in the SAP group were not significantly different from those of patients treated with TAP (*Table 2*). At 12 months’ follow-up, similar improvement of the VEINES-QOL/Sym scores compared with baseline was seen in both treatment groups (*Table 2* and *Fig. 2*). The mean change in VEINES-QOL was +9.4 (95 per cent c.i. 8.2 to 10.4) points for the SAP group and +10.8 (9.9 to 11.8) points in the TAP group (*P* = 0.157). The mean change in VEINES-Sym score was +11.6 (10.4 to 12.6) and +12.9 (12.2 to 14.2) points respectively (*P* = 0.185).

**Table 2:** Quality-of-life measurements

	SAP		TAP		P*
	Baseline	12 months	Baseline	12 months	
**Disease-specific HRQoL**		*n* = 183		*n* = 190	
ȃVEINES-QOL	43.3 (41.9, 44.7)	52.7 (51.9, 53.9)	43.0 (41.6, 44.3)	53.8 (53.3, 55.1)	0.394
ȃVEINES-Sym	41.9 (40.5, 43.3)	53.5 (52.6, 54.4)	41.3 (39.9, 42.7)	54.2 (54.0, 55.6)	0.514
**Generic HRQoL**		*n* = 186		*n* = 195	
ȃEQ-5D™	0.808 (0.779, 0.838)	0.931 (0.911, 0.950)	0.820 (0.796, 0.844)	0.939 (0.921, 0.956)	0.540
ȃEQ-VAS	77.7 (75.5, 79.5)	81.6 (79.7, 83.5)	76.3 (74.3, 78.3)	82.6 (81.0, 84.1)	0.444

Values are mean (95% c.i.). SAP, isolated ambulatory phlebectomy with or without delayed endovenous truncal ablation; TAP, thermal ablation with concomitant phlebectomy; HRQoL, health-related qualtiy of life; VEINES-QOL/Sym outcomes VEnous INsufficiency Epidemiological and Economic Study Quality of Life/Symptoms; EQ-5D™, EuroQol Five Dimensions; EQ-VAS, EuroQol visual analogue scale. VEINES-QOL/Sym outcomes were analysed in a multilevel repeated-measures model. EQ-5D™ and EQ-VAS scores were analysed using an independent *t* test. *SAP *versus* TAP at 12 months’ follow-up.

### Non-inferiority

**Fig. 3:**
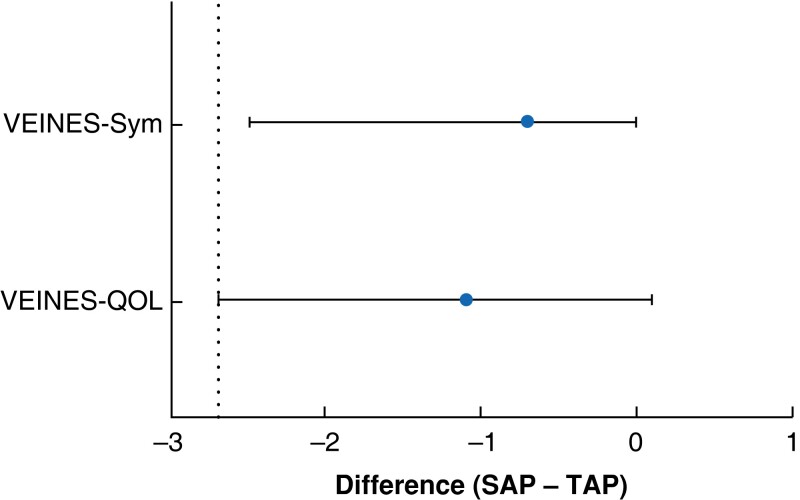
**Mean difference in VEINES-QOL/sym scores between** treatment groups, **and non-inferiority margin** Values are mean differences with 95% confidence interval; dotted line indicates non-inferiority line. VEINES-QOL/Sym, VEnous INsufficiency Epidemiological and Economic Study Quality of Life/Symptoms; SAP, isolated ambulatory phlebectomy with or without delayed endovenous truncal ablation; TAP, thermal ablation with concomitant phlebectomy.

At 12 months’ follow-up, the mean difference in VEINES-QOL scores between the SAP and TAP groups was −1.1 (95 per cent c.i. −2.7 to 0.1) points; for VEINES-Sym scores, it was −0.7 (−2.5 to 0.0) points. The non-inferiority margin, predetermined at 5 per cent of the VEINES-QOL/Sym scores at 12 months’ follow-up for the TAP group, was −2.7 points (*Fig. 3*)^20^.

### Secondary outcomes

#### Additional treatment of a refluxing saphenous trunk in SAP group at 9 months

Of all 227 patients in the SAP group, 58 (25.6 per cent) received additional truncal treatment after 9 months, either by EVLA, UGFS or radiofrequency ablation with VNUS ClosureFAST. The additional treatment was not specified for one patient. No additional truncal treatment was required in 167 patients (73.5 per cent) (*Table 3*). Two patients were lost to follow-up and it was not known if they underwent any additional truncal treatment.

**Table 3:** Additional treatments of great saphenous vein and anterior accessory saphenous vein in isolated ambulatory phlebectomy with or without delayed endovenous truncal ablation group

	No. of patients (*n* = 227)
No additional treatment	167 (73.5)
**Additional treatment**	58 (25.6)
ȃEVLA	51 (22.5)
ȃUGFS	4 (1.8)
ȃRFA	2 (0.9)
ȃOther	1 (0.4)
Unknown	2 (0.9)

Values are *n* (%). EVLA, endovenous laser ablation; UGFS, ultrasound-guided foam sclerotherapy; RFA, radiofrequency ablation.

#### Patient satisfaction and generic quality of life

**Fig. 4:**
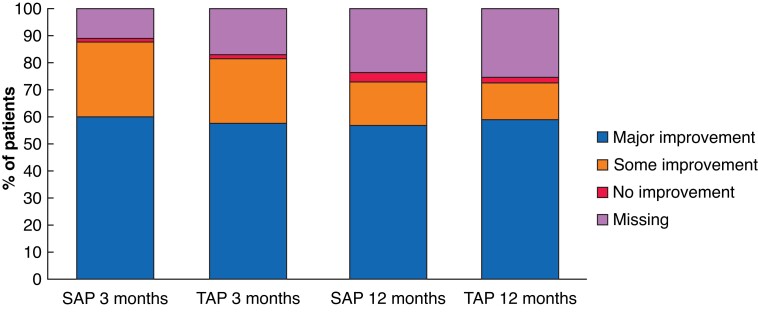
Perceived improvement after 3 and 12 months’ follow-up SAP, isolated ambulatory phlebectomy with or without delayed endovenous truncal ablation; TAP, thermal ablation with concomitant phlebectomy.

Twelve months after the initial treatment, patients in both groups were equally satisfied about their treatments. On a scale from 0 to 10, the mean satisfaction score was 8.4 (95 per cent c.i. 8.2 to 8.6) in the SAP group and 8.6 (8.4 to 8.7) in the TAP group. The majority of patients (72.7 per cent in SAP group and 72.5 per cent in TAP group) experienced major or some improvement in initial symptoms (*Table 4* and *Fig. 4*). At 12 months’ follow-up, EQ-5D™ and EQ-VAS scores had improved compared with baseline in both groups, but these changes were minimal (*Table 2*).

**Table 4:** Patient satisfaction and perceived improvement after treatment

	SAP	TAP	P*
**3 months’ follow-up**	*n* = 202	*n* = 197	
ȃPatient satisfaction (score 0–10), mean (95% c.i.)	8.2 (8.0, 8.3)	8.3 (8.2, 8.4)	
ȃPerceived improvement			0.793
ȃȃNo improvement at all	4 (1.8)	5 (2.1)	
ȃȃSome improvement	63 (27.8)	56 (23.6)	
ȃȃMajor improvement	135 (59.5)	136 (57.4)	
**12 months’ follow-up**	*n* = 173	*n* = 177	
ȃPatient satisfaction (score 0–10), mean (95% c.i.)	8.4 (8.2, 8.6)	8.6 (8.4, 8.7)	
ȃPerceived improvement			0.563
ȃȃNo improvement at all	8 (3.5)	5 (2.1)	
ȃȃSome improvement	36 (15.9)	33 (13.9)	
ȃȃMajor improvement	129 (56.8)	139 (58.6)	

Values are *n* (%), unless indicated otherwise. SAP, isolated ambulatory phlebectomy with or without delayed endovenous truncal ablation; TAP, thermal ablation with concomitant phlebectomy. Patient satisfaction scores were analyzed in a multilevel repeated-measures model. *χ^2^ test.

#### Venous Clinical Severity Score and anatomical success

The mean VCSS of patients in the SAP group was higher after 12 months than that in the TAP group: 1.86 (95 per cent c.i. 1.55 to 2.18) and 1.22 (1.01 to 1.43) respectively (*P* = 0.001). The mean improvement in VCSS at 12 months compared with baseline was −4.1 (95 per cent c.i. –4.5 to −3.8) for the SAP group, less than that noted for the TAP group, for which the mean improvement was −4.8 (−5.1 to −4.5).

**Fig. 5:**
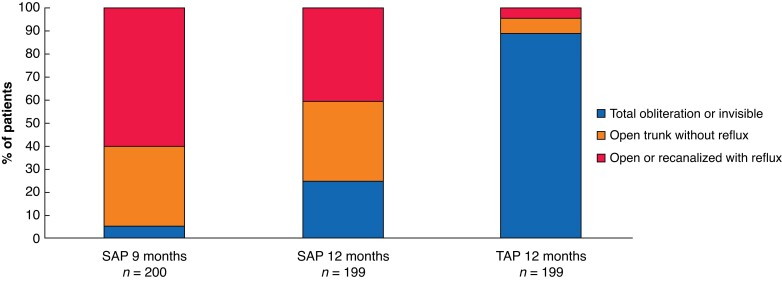
Anatomical success, according to duplex ultrasonography after 9–12 months SAP, isolated ambulatory phlebectomy with or without delayed endovenous truncal ablation; TAP, thermal ablation with concomitant phlebectomy. *P* <0.001 for anatomical success at 12 months (χ^2^ test).

At 12 months’ follow-up, the anatomical treatment success rate at the level of the GSV or AASV was higher in the TAP group: 177 patients (88.9 per cent) had a total obliteration, 13 (6.5 per cent) had an open trunk without reflux, in 7 patients (3.5 per cent) the trunk was completely recanalized with reflux, and in 2 patients (1.0 per cent) it was partially recanalized with reflux. In the SAP group, anatomical treatment success at the level of the saphenous trunk was achieved in 115 patients (57.8 per cent) (48 patients (24.1 per cent) had a totally obliterated or invisible saphenous trunk, and 67 (33.7 per cent) had a completely open trunk without reflux); 84 patients (42.2 per cent) still had a refluxing trunk (*Fig. 5* and *Table 5*). Of the 48 patients with a totally obliterated or invisible trunk, 37 had received additional treatment at 9 months. The saphenous trunk was not visible in the other 11 patients.

**Table 5:** Anatomical outcomes

	SAP	TAP	P*
**9 months’ follow-up**	*n* = 200	–	–
ȃTotal obliteration or invisible	10 (5.0)	–	
ȃOpen trunk without reflux	69 (34.5)	–	
ȃOpen or recanalized with reflux	121 (60.5)	–	
**12 months’ follow-up**	*n* = 199	*n* = 199	<0.001*
ȃTotal obliteration or invisible	48 (24.1)	177 (88.9)	
ȃOpen trunk without reflux	67 (33.7)	13 (6.5)	
ȃOpen or recanalized with reflux	84 (42.2)	9 (4.5)	
**SFJ reflux at 12 months’ follow-up**	*n* = 191	*n* = 192	0.405*
ȃNo	189 (99.0)	187 (97.4)	
ȃYes	2 (1.0)	5 (2.6)	

Values are *n* (%). SAP, isolated ambulatory phlebectomy with or without delayed endovenous truncal ablation; TAP, thermal ablation with concomitant phlebectomy; SFJ, saphenofemoral junction. *χ^2^ test.

#### Cost-effectiveness

Treatment costs in the SAP group were €1030 (95 per cent c.i., 885 to 1092) per patient including the cost of additional DUS, and €1660 (1544 to 1779) in the TAP group, The SAP treatment was on average €630 (571 to 782) less costly than the TAP treatment.

**Fig. 6:**
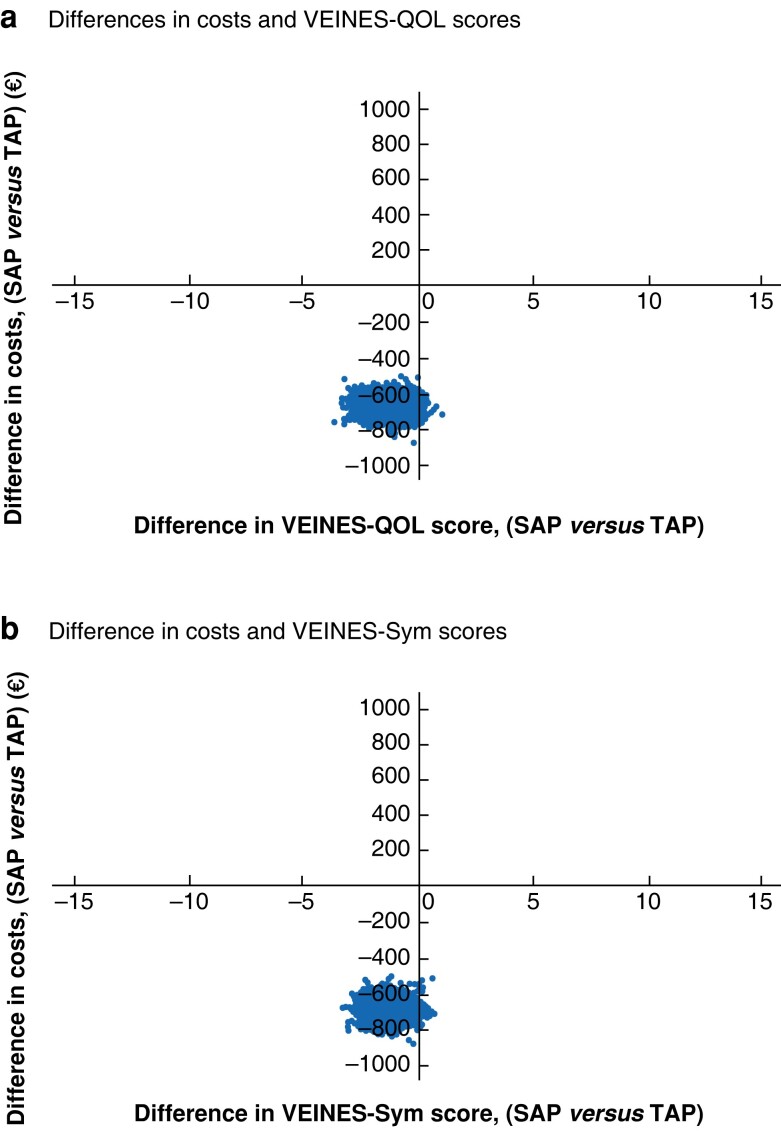
Cost-effectiveness planes for VEINES-QOL (a) and VEINES-Sym (b). VEINES-QOL/Sym, VEnous INsufficiency Epidemiological and Economic Study Quality of Life/Symptoms; SAP, isolated ambulatory phlebectomy with or without delayed endovenous truncal ablation; TAP, thermal ablation with concomitant phlebectomy.

*Fig. 6* shows cost-effectiveness displayed in a cost-effectiveness-plane, where each point represents an outcome of one bootstrap iteration. Most points are in the lower-left quadrant, showing lower costs per patient but also a slightly lower HRQoL outcome in the SAP group.

#### Complications

Complications were mostly minor and similar in both groups. A total of 25 patients in the SAP group developed complications, consisting of postoperative bleeding (4), SVT (10), and hyperpigmentation (11). In TAP patients, 22 complications occurred, consisting pulmonary embolism in 1 patient and minor complications in 21: postoperative bleeding (4), postoperative wound infection (1), nerve damage (1), SVT (5), and hyperpigmentation (10). None of the patients developed postoperative deep venous thrombosis.

## Discussion

In the SAPTAP trial, treatment with SAP was not inferior to treatment with TAP for patients with an incompetent GSV or AASV and one or more incompetent tributaries. This conclusion is based on the finding that the 95 per cent confidence intervals of the difference in VEINES-QOL/Sym scores between the SAP and TAP groups fell within the predetermined non-inferiority margin. This finding is supported by analysis of HRQoL outcomes (VEINES-QOL/Sym, EQ-5D™, and EQ-VAS scores). Twelve months after the first treatment, HRQoL outcomes were similar for the two procedures, and had improved similarly in both groups compared with baseline. Patient-reported satisfaction regarding the treatment and subjective improvement of symptoms were similar in both groups after 12 months.

Treating patients with SAP instead of TAP has several advantages. First, only 25.6 per cent of those in the SAP group required additional treatment 9 months after ambulatory phlebectomy. Additional truncal treatment was not required in 73.5 per cent of the patients as they had no symptoms (with or without truncal reflux) at 9-month follow-up. Thus, three-quarters of the patients were relieved of symptoms after one instead of two treatments. Second, the GSV/AASV was spared in these 73.5 per cent of patients by being treated with SAP. Sparing the saphenous trunk may result in abolishment of reflux, owing to reduction of blood volume in the trunk after isolated phlebectomy. Therefore, it is given the opportunity to regain its function as one of the main veins of the superficial venous system. It may even be used as a bypass graft in later life. However, when selecting patients for SAP treatment, they should be informed about the potential need for additional truncal treatment. This extra treatment would cost patients an extra visit to the hospital, as well as an extra day of absence from work or other duties.

The findings of the SAPTAP trial in terms of anatomical success are comparable to those of previous studies of the ASVAL method. After 1-year follow-up, GSV/AASV reflux was eliminated in 57.8 per cent of patients in the SAP group, whereas symptoms had decreased in 72.7 per cent of the participants. These outcomes are comparable to those of a prospective observational study^6^, in which 50 and 66 per cent of the patients had elimination of GSV reflux and symptoms respectively. Other prospective studies also described abolishment of truncal reflux in 60 per cent of patients 6 months after the initial treatment with the ASVAL method^10,12,21^.

Anatomical success rates after 12 months were clearly higher after TAP than SAP (95.5 *versus* 57.8 per cent respectively). However, it should be noted that the severity of symptoms often does not correlate with clinical and DUS findings in patients with CVD^22–27^. Therefore, treatments for CVD should mainly aim at improving quality of life. In the present RCT, as in previous studies^24,27–32^, different management strategies for treating superficial venous incompetence have been proven to be beneficial to quality of life. Whether these positive effects of SAP will prove to be long-lasting is a subject for future research.

From a healthcare-related costs perspective, treatment with SAP was cheaper, saving €630 per patient compared with treatment with TAP. In the Netherlands, around 25 000 EVLA procedures are performed per year for incompetent GSVs or AASVs^33^. In a certain proportion of these, concomitant phlebectomies are undertaken (TAP), conforming with the Dutch guideline on varicose vein treatment^34^. However, as Dutch healthcare insurance companies are not currently covering concomitant phlebectomy of tributaries, if performed simultaneously with EVLA or an alternative ablation technique, the exact number of combined procedures including phlebectomies such as TAP per year is unknown. Only the total number of thermal or non-thermal ablation procedures is registered in the Dutch healthcare database^33^. For instance, in 2019, 24 850 EVLA procedures were registered. As there are no direct data available, the proportion of EVLA procedures in which concomitant phlebectomy has been performed can only be estimated approximately. It was decided to base this estimation on a meta-analysis^4^, which reported that 36 per cent of the patients with GSV reflux need additional treatment for refluxing tributaries. In view of these findings, it could be assumed that concomitant phlebectomy would have been performed in at least in 30 per cent of EVLA procedures registered in 1 year in the Netherlands. As an example, for the year 2019, 30 per cent would entail a total of 7455 TAP treatments. Based on the findings of the present trial, replacing the latter number of procedures by a SAP treatment could have resulted in a cost saving of €630 per patient or €4 696 760 for the healthcare insurance companies in 1 year. The current Dutch healthcare insurance reimbursement criteria are based on the descending pathophysiological theory of the origin of varicose veins; reimbursement for varicose vein treatment is only available for patients with an incompetent SFJ or incompetent perforating veins. Based on the findings of the present study, as well as other studies^6–12^ supporting the ascending pathophysiological theory, the authors’ advice would be to reconsider the reimbursement criteria for patients with symptomatic varicose veins.

This study has shown the non-inferiority of SAP compared with TAP, without application of any selection criteria for SAP. Further research should determine these selection criteria, ideally leading to a smaller number of patients needing an additional EVLA during follow-up.

The present study has several limitations. First, the number of patients included for analysis was slightly smaller than originally calculated: a sample size of 472 patients was required and only 464 patients were analysed. A second limitation is the relatively short follow-up time of 1 year. Third, although the primary outcome was clearly defined, multiple secondary outcomes are reported. The findings among secondary outcomes were mostly negative and in accordance with each other, suggesting that multiple comparison bias did not affect the results. Additionally, the SAP group had an additional visit at 9 months, which might have influenced the quality-of-life outcomes. Furthermore, it should be acknowledged that the extra burden for patients undergoing SAP—having to return to the clinic for a supplementary visit after 9 months, including DUS, an additional intervention in 25.6 per cent, and the related absence of work or other activities—has not been taken into account. Indirect costs for both treatment groups were not considered in this study. Moreover, treatment costs were based on prices determined by the health insurance companies, and not on actual costs in different hospitals. Therefore, these costs are only an approximation of the real healthcare costs. Finally, the choice of the non-inferiority margin is somewhat arbitrary. This is always true in non-inferiority studies, but an additional element here is that the VEINES-QOL/Sym is calibrated to have a mean of 50 in any particular sample. This means that differences in scores are relative to the mean quality of life in the sample as a whole, even at the level of individual patients. As a consequence, a 5 per cent difference represents a slightly larger difference in quality of life in samples with relatively healthy patients. The non-inferiority margin was set based on the maximal acceptable loss of HRQoL. Because the VEINES-QOL/Sym score is a relative score, and does not have an absolute value, the absolute meaning of this loss in HRQoL is not known.

The SAPTAP trial shows that HRQoL after one year follow-up is equal for patients treated with SAP and with TAP. Treatment with SAP with or without delayed endovenous truncal ablation is a cost-effective alternative for TAP. Treatment with SAP not only resulted to be less expensive, but patients could also avoid an additional saphenous trunk treatment in 73.5% of the cases, based on a follow-up period of one year.

## Data Availability

The data that support the findings of this study are available from the corresponding author upon reasonable request.

## Collaborators

Members of the SAPTAP Group: E.R.Y. Scheerders, S.A.S. Hamann, W.S.J. Malskat, M.G.R. de Maeseneer, R.R. van den Bos (Erasmus MC, Rotterdam, The Netherlands); S.K. van der Velden (MohsA Clinics Skincenter, Eindhoven, The Netherlands); L. de Mik (Isala, Zwolle, The Netherlands); M.T.W. Gaastra (Center Oosterwal, Alkmaar, The Netherlands); S. Koppen (St Antonius Hospital, Utrecht, The Netherlands); K.P. De Roos (DermaPark, Uden, The Netherlands); N.H. Shadid (Medical Center Haaglanden, Leidschendam, The Netherlands); O. Wolff (Mohs Clinics, Dordrecht, The Netherlands).

## Data sharing

Relevant deidentified data will be available on reasonable request.
